# The potential of polygenic scores to improve cost and efficiency of clinical trials

**DOI:** 10.1038/s41467-022-30675-z

**Published:** 2022-05-25

**Authors:** Akl C. Fahed, Anthony A. Philippakis, Amit V. Khera

**Affiliations:** 1 Division of Cardiology and Center for Genomic Medicine, Massachusetts General Hospital, Boston, MA USA; 2 Program in Medical and Population Genetics, Broad Institute of Harvard and MIT, Cambridge, MA USA; 3 Department of Medicine, Harvard Medical School, Boston, MA USA; 4 Data Sciences Platform, Broad Institute of MIT and Harvard, Cambridge, MA USA; 5 Eric and Wendy Schmidt Center, Broad Institute of MIT and Harvard, Cambridge, MA USA; 6 Verve Therapeutics, Cambridge, MA USA

**Keywords:** Personalized medicine, Predictive markers, Prognostic markers, Randomized controlled trials

## Abstract

Polygenic scores can identify individuals with high disease risk based on inborn DNA variation. We explore their potential to enrich clinical trials by identifying individuals based on higher risk of disease (‘prognostic enrichment’), or increased probability of benefit (‘predictive enrichment’).

Clinical trials typically study rates of disease in participants randomized to a placebo or a given intervention, serving two primary purposes—first, to provide ‘gold standard’ evidence of efficacy and safety needed to obtain regulatory approval; and second, to demonstrate adequate benefit to convince clinicians and payers to use the drug within clinical practice. Because such trials for common diseases often require tens of thousands of participants followed for several years, the typical cost is $350 million, out of reach for all but the largest pharmaceutical companies or governmental agencies^1^.

One important approach to increase clinical trial efficiency is to selectively enroll participants based on clinical or molecular characteristics^2^. Guidance from the U.S. Food and Drug Administration outlines two distinct conceptual approaches for enrichment. The first, termed ‘prognostic enrichment,’ aims to increase statistical power—and thus decrease sample size and cost—by increasing the proportion of patients likely to demonstrate disease onset or progression. Taking COVID-19 vaccine trials as an example, Moderna and other sponsors selectively enrolled participants in areas where the virus was rapidly spreading to more quickly demonstrate benefit^3^. For a new cholesterol-lowering therapy designed to prevent heart attack and stroke, the pivotal trial enrolled only those with preexisting cardiovascular disease based on data that the event rates in these individuals is much higher^4^. The second, termed ‘predictive enrichment,’ aims to enroll participants who are more likely to have an outsized benefit to the trial intervention. Demonstration that patients whose lung cancer contained specific gain-of-function mutations in the target of an inhibitor of this receptor’s signaling respond to treatment, while those without such mutations do not, inspired a new era in oncologic development where predictive enrichment using molecular profiling has substantially reduced development cost and duration^2,5^.

Despite the frequent use of enrichment strategies, clinical trials still often fail to achieve their aim of allowing the intervention to gain regulatory approval and adoption in clinical practice. These (costly) failures are particularly common when low event rates preclude the preferred trial design or the existing standard of care is already good, thus making the demonstration of a meaningful improvement with a new drug more challenging. For conditions such as Alzheimer’s dementia, enrollment of participants late in the disease process—which aims to increase event rates via prognostic enrichment—is often cited as a potential reason for the failures that have occurred even when the therapeutic target is believed to be pathophysiologically sound, as was the case for an antibody designed to clear amyloid plaques from the brain^6,7^. In cardiovascular disease, a powerful cholesterol-lowering medicine reduced the frequency of clinical events from 11.8 to 10.8% compared to placebo, achieving its primary endpoint with a compelling degree of statistical confidence (*p* = 0.004), but this effect size was deemed inadequate to justify pursuing its commercialization^8^.

Given these challenges in clinical trial design and execution, are genetic enrichment strategies using ‘polygenic scores’ worthwhile to consider?

The traditional approach to genetic risk stratification has focused on identifying the small subset of the population with rare monogenic mutations that substantially increase risk via disruption of a specific biologic pathway. More recently, polygenic scores—which instead consider the cumulative impact of many common DNA variants scattered across the genome—have gained traction as a promising approach with relevance for much larger subsets of the population. Initially proposed for applications in plant and animal breeding, newer generation polygenic scores have considerable predictive capacity across a range of important common diseases^9–11^. This stratification allows for the identification of individuals (as early as birth) whose inborn DNA variation places them on a markedly accelerated trajectory of disease onset. For coronary artery disease, we demonstrated that up to 8% of the population inherits triple the normal risk based on genetic variation alone, and these high-risk individuals cannot be reliably identified with traditional risk factors or family history^10^.

*Post hoc* analyses of clinical trials involving cholesterol-lowering therapies for cardiovascular disease have suggested that polygenic scores hold promise as a powerful enrichment strategy. Among healthy individuals randomized to statin or placebo to prevent cardiovascular disease, those with the highest polygenic score demonstrated the greatest benefit^12,13^. This benefit was related to both prognostic enrichment—the rates of developing heart disease in the placebo group was 19.6% for those in the top quintile of the score versus 12.9% in all others—*and* predictive enrichment, where a 44% relative risk reduction was noted for those with high score versus only 24% in the remainder of the participants^13^.

This observation from statin trials was later extended to two trials focused on preventing a second cardiovascular event in those with existing disease using powerful (and expensive) new injectable medications, where those with the highest polygenic score again derived the greatest benefit due to both prognostic and predictive enrichment^14,15^. This analysis suggests that—had it been possible to predict this enrichment in advance—the trials could have successfully demonstrated benefit with substantially fewer participants (Fig. 1). In this specific case, we estimate that a trial that enrolled only those participants in the top quintile of the polygenic score might have required only 2360 participants—a greater than 90% reduction from the 27,564 studied—and demonstrated a 31% relative risk reduction as compared to the 20% observed in the overall trial population. For a drug class that faced post-approval access challenges, initial commercialization for a subset of the population who derived greater benefit may have enhanced clinical uptake, perceived cost-effectiveness, and overall public health impact.

**Fig. 1:**
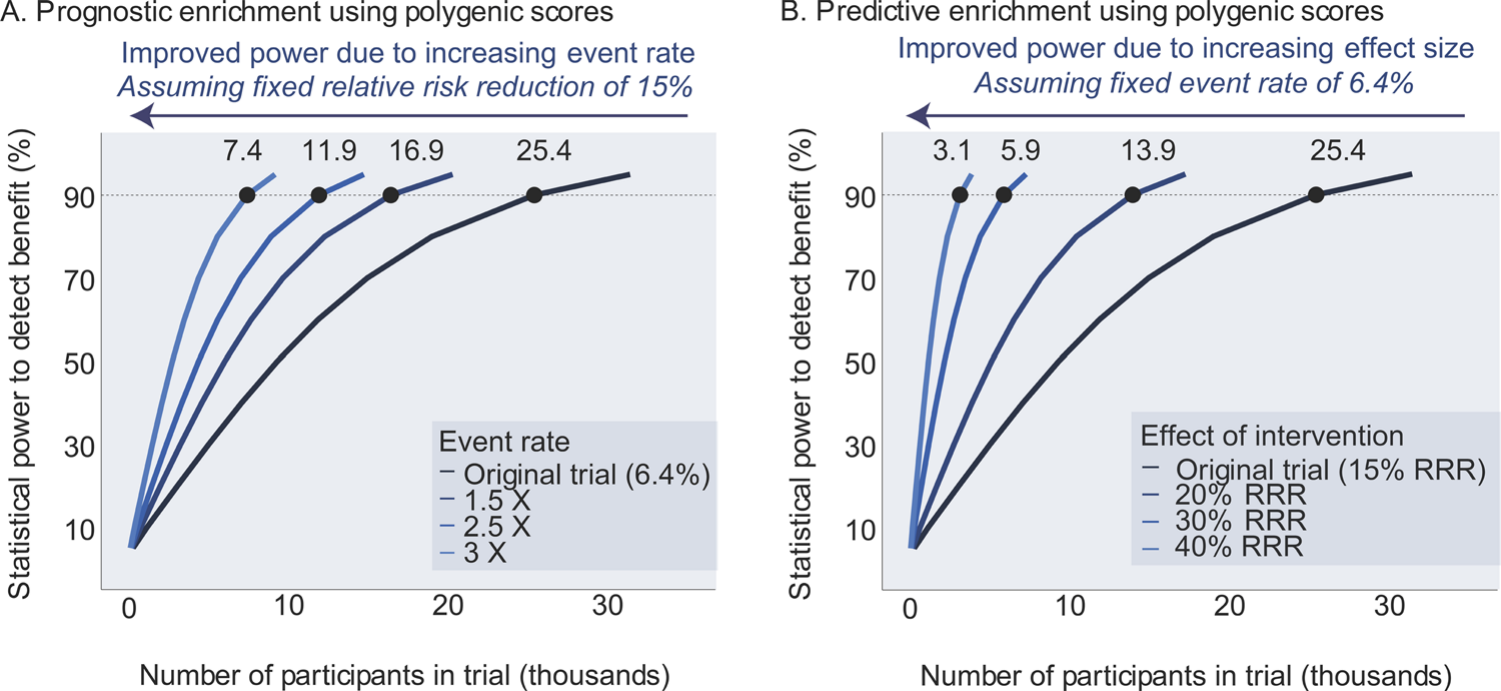
Power and sample size estimation using prognostic or predictive model for polygenic score enrichment. The FOURIER clinical trial randomized 27,564 patients with cardiovascular disease to a placebo or evolucumab, a cholesterol-lowering therapy, and followed patients for a median of 2.2 years^4^. This trial design was based on a power calculation that predicted an event rate of ~6.4% in the control arm and a relative risk reduction (RRR) of 15%^28^. We used these data to model power calculations using polygenic score enrichment under either of two models. **A** With prognostic enrichment (increasing event rates beyond the 6.4% in the original trial), a polygenic score enrichment improves statistical power to detect a benefit despite a fixed effect size (relative risk reduction of 15%); **B** with predictive enrichment (increasing effect size of intervention beyond the 15% RRR in the original trial), a polygenic score enrichment improves power with a fixed event rate in the placebo arm of 6.4%. The dashed line in both panels denotes 90% power to detect a statistical benefit, a threshold commonly used in trial design. Using polygenic scores to enrich clinical trials could markedly improve power and reduce the number of participants needed by increasing event rates (“prognostic enrichment”) and/or increasing the effect size (“predictive enrichment”).

We believe that polygenic risk estimation will play an important role in the future of clinical medicine, enabling targeted screening or prevention strategies to overcome inherited predisposition, and warrants consideration as an enrichment strategy for clinical trials as well. Although the potential requirement of a genetic test as an inclusion criterion for a given trial creates a potential hurdle to recruitment, this has become common within clinical medicine for use cases ranging from targeted cancer therapies, drugs for cystic fibrosis that work only among those with a given genetic mutation in the *CFTR* gene, or a potent fish oil formulation that is approved only for those with high circulating triglyceride levels^2,16,17^. Compelling use cases might include primary prevention trials where traditional approaches would require a clinical trial that is intractable owing to the very large sample size and long follow-up that would be necessary to show benefit. Beyond conditions such as Alzheimer’s dementia discussed above, an additional public health need relates to nonalcoholic fatty liver disease—which affects up to 20% of the world’s population and is the leading risk factor for liver cirrhosis or cancer—but has been challenging to conduct trials for since only a small fraction of afflicted individuals progress to more advanced disease in a given year^18^. We and others have recently developed polygenic scores for this condition, laying the scientific foundation for a new generation of trials that incorporate genetic enrichment strategies^19^.

Alongside the considerable (and warranted) enthusiasm for the use of polygenic scores to meaningfully enhance clinical development, several potential barriers warrant discussion. First, the predictive capacity of a polygenic score is limited by heritability (proportion of risk explained by common DNA variants) and scores may not have adequate ability to stratify risk for some conditions^20^. Second, although in principle polygenic scores can be assessed for less than $100 U.S. dollars, few patients or healthcare systems currently offer them clinically, posing a logistical challenge for trial enrollment or medication prescribing. Third, current polygenic scores are typically associated with increased risk across all ancestries, but with an effect size that is highest in those of European ancestry (primarily due to lack of adequate training data in other groups)^21,22^. Fourth, most scores developed to date are based on case-control datasets for a given disease—additional work is needed to determine whether the genetic basis of disease progression meaningfully differs from disease onset and whether ‘pathway-specific’ scores may provide more reliable predictive enrichment^23,24^. Fifth, an approach that integrates polygenic risk with additional rare genetic or non-genetic factors such as clinical or biomarker concentrations is likely to outperform strategies based on a polygenic score alone, but few such algorithms have been developed to date^25,26^. Sixth, the regulatory guidelines surrounding polygenic score use in clinical development have not been fully articulated and scores are likely to evolve over time due to a lack of accepted standards to evaluate performance and reproducibility—increasing the risk of a sponsor obtaining an approved drug label with a given score. Seventh, most investigations of utilizing polygenic scores in clinical trials are from post hoc analyses, but prospective implementation may still face logistical and scientific challenges that would need to be solved.

Despite potential barriers, the high cost of clinical trials has emerged as arguably the single biggest barrier to the development of innovations that may well have substantial public health benefit—and potential strategies to meaningfully alter this landscape mandate serious consideration^27^. As observed in trials of cholesterol-lowering therapies, polygenic scores hold the potential to enable substantial predictive or prognostic enrichment and could have a deep impact on enabling a new era in clinical development.
